# Situated drinking: The association between eating and alcohol consumption in Great Britain

**DOI:** 10.1177/14550725231157222

**Published:** 2023-02-21

**Authors:** Alan Warde, Alessandro Sasso, John Holmes, Monica Hernández Alava, Abigail K. Stevely, Petra S. Meier

**Affiliations:** School of Social Sciences, Sustainable Consumption Institute, AMBS, 5292 University of Manchester, Manchester, UK; Sheffield Alcohol Research Group, School of Health and Related Research, 7315 University of Sheffield, Sheffield, UK; Sheffield Alcohol Research Group, School of Health and Related Research, 7315 University of Sheffield, Sheffield, UK; School of Health and Related Research, 7315 University of Sheffield, Sheffield, UK; Sheffield Alcohol Research Group, School of Health and Related Research, 7315 University of Sheffield, Sheffield, UK; MRC/CSO Social and Public Health Sciences Unit, 3526 University of Glasgow, Glasgow, UK

**Keywords:** alcohol consumption, class, drinking with meals, domestic practices, food, gender, Great Britain

## Abstract

**Aims:** This paper examines the co-occurrence of drinking alcohol and eating in Great Britain. Applying a practice-theoretical framework, it attends primarily to the nature and characteristics of events – to social situations. It asks whether drinking events involving food are significantly different from those without, whether differences are the same at home as on commercial public premises, and whether differences are the same for men and women. The focus is especially on episodes of drinking with meals at home, an infrequently explored context for a substantial proportion of contemporary alcohol consumption. **Data:** Employing a secondary analysis of commercial data about the British population in 2016, we examine reports of 47,645 drinking events, on commercial premises and at other locations, to explore how eating food and consumption of alcoholic beverages affect one another. Three types of event are compared – drinking with meals, with snacks, and without any food. Variables describing situations include group size and composition, temporal and spatial parameters, beverages, purposes, and simultaneous activities. Basic sociodemographic characteristics of respondents are also examined, with a special focus on the effects of gender. **Results:** Behaviours differ between settings. The presence of food at a drinking episode is associated with different patterns of participation, orientations, and quantities and types of beverage consumed. Gender, age, and class differences are apparent. **Conclusions:** Patterns of alcohol consumption are significantly affected by the accompaniment of food. This is a much-neglected topic that would benefit from further comparative and time series studies to determine the consequences for behaviour and intervention.

This paper examines the characteristics of events involving the co-occurrence of drinking alcohol and eating (drinking-*and*-eating^i^). Since 2000, a significantly greater proportion of the alcohol consumed in Britain has been drunk at home (British Beer and Pubs Association (BBPA), 2018). It seems likely that more drinking occasions include food, particularly as accompaniments to meals. The habit of dining out for pleasure, which increased very significantly in the later part of the 20th century, also provides opportunities for more drinking with food (Warde et al., 2020b). Yet the specific association between eating and drinking alcohol is rarely studied. Eating and drinking are typically examined independently of one another, partly a result of the academic division of labour but largely because they serve different policy communities. Consequently, scholarly coverage of their interaction is very uneven and drinking-and-eating occasions are poorly understood.

Part of a wider study, “Social Practices in Alcohol Research Collaboration” (SPARC), based on a secondary analysis of a commercial database about British drinking since 2000, this paper examines the reports of 47,645 events in 2016, on licensed premises (“on-trade”) and in other locations (“off-trade”). It explores how eating food during a drinking episode is associated with the social arrangements of the event and the alcohol consumed. Three substantive empirical questions are addressed:

- Are drinking events involving food significantly different from those without?
- Are differences the same at home as on commercial public premises?
- Are differences the same for men and women?

Answering these questions helps to provide a systematic picture of the nature of drinking events in the UK today, improves our understanding of “moderate” and “ordinary” drinking, and identifies a concealed aspect of household meals. Perhaps the normalisation of drinking at home is associated with a propensity to drink with meals. A better understanding of current practice can have both scholarly and policy benefits.

## Context

Historical accounts of drinking establishments in Britain offer an adequate understanding of the institutional framework of drinking-and-eating in public settings. Food was available in pubs and taverns serving business districts in large cities, and hotels provided both elaborate food and drink from the mid-19th century (Burnett, 1999; Lane, 2018). In residential areas, the public house was the major venue for drinking, sometimes selling snacks but typically not providing any food (Burnett, 1999). Restaurants also supplied both meals and alcohol, but they were less common than now, the practice of dining out for pleasure having spread from an elite minority to a much wider population only late in the 20th century (Burnett, 2004; Warde et al., 2020b). Cafes and works canteens were rarely licensed. Drinking and eating in public were generally served by separate businesses.

Drinking in domestic settings is less well understood. An important change in the last 50 years has been the growth of off-trade sales and a tendency for people to drink alcohol more frequently at home. Beverages purchased to drink at home increased from 53% to 67% of the total between 2000 and 2017 (BBPA, 2018, Tables B9 and B10). In terms of volume of alcohol consumed, off-trade consumption was a mere 13% greater than the on-trade in 2000, but by 2016 it was more than double (133% greater) (BBPA, 2018). Previously, drinking at home was uncommon and meals were rarely accompanied by alcohol. Warren (1958), for example, shows that drinking with meals in the 1950s was infrequent and was very rare among the working class who typically drank tea with meals (Vogler, 2020). Now more people drink alcohol with meals, yet almost no research exists to capture the nature of that experience, what it might mean, how levels of consumption are regulated, and how it might express social relationships or indicate social status. Studies specifically of household meals almost never record what is drunk, despite alcoholic accompaniments signifying the format and rank of the meal and the status and relationships of the diners. In addition, the difference between a meal and a snack might be symbolically significant and be differently positioned.

Many factors affect the likelihood of alcohol being present at a meal including family socialisation, religious commitment, and national culture. In the European perspective, Britons drink more frequently at home, both with and without meals, and in public venues such as pubs and restaurants than most other EU countries (Reducing Alcohol Related Harm (RARHA), 2016). A survey by RARHA (2016, p. 119) reported that in 2013 about 35% of UK adults who drink alcohol had consumed alcohol with a meal at home once a week or more often during the previous 12 months; the UK was fifth highest out of 19 EU countries surveyed, behind Bulgaria, Portugal, Catalonia, and Italy.^ii^ Britain was similarly ranked for frequency of drinking with meals on commercial premises, including in pubs and restaurants.

Whether alcohol is accompanied by food affects what is drunk, how much, for what reason, and with whom. Yet reference in the alcohol studies literature to drinking with food is very sparse and mostly incidental. Market research on the purchase of wine supplies some clues but mostly takes for granted that meals are the principal context for drinking wine and therefore investigates no further (e.g., Leifman, 2002). Hupkens et al.'s (1993) study of EU countries in 1988 finds less drinking with meals in northern Europe. Jayne et al. (2008, p. 93) note that Britons consider drinking with meals as “civilised” and “European”. Ritchie (2007) shows that wine had become “the drink of choice” at domestic and commercial meals by 2005. Meanwhile, connoisseurs and columnists give advice about which drinks and foods “match” (e.g., Welch and Tominc, 2019). Jani et al. (2021) argue that alcohol accompanying meals has less harmful effects, especially with respect to wine, suggesting that meals may be a context for drinking in moderation. With regard to ordinary and moderate consumption, we know comparatively little, mostly because the scholarly focus is largely on the harmful effects of excessive amounts of alcohol (though see Ally et al., 2016). As Thurnell-Read (2020) observes, attention to situations of intoxication dwarfs the much more frequent episodes where smaller amounts of alcohol are consumed. In such cases, we might consider the drinking a complementary or a subsidiary activity, whether or not the event occurs at home or on commercial premises.

What is to be eaten and drunk is often a function of who is present. Company at home is most likely to be other household members, although hospitable domestic events engage friends and non-resident kin. The gender composition of groups also matters. Leifman's (2002) study of six European countries in 2000 notes that women are more likely than men to consume alcohol with meals, while Pratten and Carlier (2010) show that men in a sample in northwest England drink wine with meals and in the company of family but infrequently otherwise.

The analysis is approached in the terms of theories of practice (Nicolini, 2012; Schatzki et al., 2001; Warde, 2016). A meso-level approach, the foundational assumption is that participants adjust to their environment through being attuned to the norms of the practice, being responsive to the cues transmitted by the structuring of the situation, and having an inclination to conform to the shared and common purposes of relevant others. The social context – the company present, the time of day, the location, mutually agreed purposes, and material and infrastructural props – conjures up a mutual understanding of norms and conventions of behaviour appropriate to the social setting and constraining the amount and types of food and drink consumed. The explanation is configurational rather than causal, the features of the setting and the behaviour of interest being viewed as mutually dependent.

A case study in the intersection of the practices of drinking and eating, this paper addresses a thorny theoretical issue. People engage in several practices at the same time and simultaneous engagement may affect how each is done. Looking at situated activity systematically is valuable because drinking with a meal may modify the consumption of both alcohol and food. The survey data employed facilitate inquiry into how activity is situated because the primary focus is discrete events that direct attention to behaviour in context rather than the agency of the individual.

The next section describes in more detail the data and the methods used for their analysis. Section 3 presents the basic findings, examining in turn off-trade and on-trade events and the differences between them, with consideration given to gender and class differences. The discussion section focuses on characteristic aspects of meal, snack and drink-only events, speculating about the nature of “normal” drinking, gender differences, and drinking-and-eating as practical performance.

## Data and methods

The Alcovision survey, carried out annually by the market research company Kantar, recruits a quota sample of 30,000 participants representative of the adult population of Great Britain. Its primary purpose is to provide detailed marketing information to the alcohol and related industries. Participants complete a retrospective online diary of their drinking activity over a seven-day period. Alcovision has a complex multilevel data structure capturing information at three levels. Drink-level information describes the characteristics of beverages reported by the respondents (e.g., type of drink, brand, and measure). Occasion-level information describes the characteristics of events, such as location, starting time, and type of venue. Individual-level information refers to personal characteristics of the respondent invariant between drinking occasions (e.g., gender, household composition, etc.). The focus of this study is on occasion and individual characteristics. The variables included in the analysis are described briefly below. Tables 1 and 2 show the values of the most relevant variables, with the full list presented in the online Appendix (Tables A1 and A2). The appendix also includes information about sample weighting and robustness checks and gives additional detailed information about gender variation (Tables A3 and A4).

**Table 1.** Summary statistics by food choice (meal consumed, snack only, no food) – off-trade occasions.

Discrete variables				
Food choice	Meal	No food	Snack	All
*Individual characteristics*				
Male	.550	.643	.601	.592
Female	.450	.357	.399	.408
Social class AB	.369	.294	.332	.336
Social class C1	.206	.207	.189	.203
Social class C2	.198	.219	.203	.206
Social class DE	.228	.280	.276	.255
Household income (<20k)	.229	.290	.279	.259
Household income (20k–35k)	.273	.280	.284	.278
Household income (20k–55k)	.253	.214	.200	.229
Household income (>55k)	.137	.122	.145	.133
Full-time worker	.454	.479	.478	.467
Other employment status	.546	.521	.522	.533
Children in the household	.261	.298	.335	.288
*Gender composition of the drinking occasion*				
Male alone	.085	.201	.156	.138
Female alone	.055	.085	.076	.069
Male pair	.047	.085	.058	.062
Female pair	.034	.038	.039	.036
Mixed pair	.393	.326	.320	.356
Male group	.024	.032	.033	.029
Female group	.019	.018	.026	.02
Mixed group	.374	.233	.336	.318
*Companionship while drinking*				
With family	.285	.147	.191	.220
With friends	.190	.154	.253	.190
With partner	.514	.409	.444	.465
With other	.025	.019	.027	.023
*Mood of the occasion*				
Wind down	.348	.401	.340	.365
To have time for themselves	.189	.166	.174	.178
*Duration of the occasion (h)*				
Duration (<1)	.305	.378	.289	.327
Duration (1–2)	.438	.471	.469	.455
Duration (>2)	.237	.151	.242	.218
*Alcohol consumption (units)*				
0–2	.144	.237	.197	.186
2–3	.223	.209	.193	.212
3–5	.133	.156	.134	.141
5–12	.330	.290	.310	.313
12–20	.097	.071	.100	.089
>20	.073	.037	.066	.059
*Main drinking beverage*				
RTD	.016	.018	.020	.018
Spirits	.155	.263	.242	.209
Cider	.114	.126	.150	.125
Wine	.458	.230	.256	.342
Beer	.256	.363	.332	.307
*Start time of the drinking occasion*				
Morning	.012	.027	.022	.019
Lunch	.063	.037	.062	.054
Afternoon	.184	.129	.144	.158
Evening	.713	.655	.664	.684
Night	.028	.152	.108	.086
*Day of the week*				
Weekdays	.385	.418	.382	.396
Weekend	.422	.438	.458	.434
Sunday	.193	.144	.160	.170
*Type of the occasion (off-trade)*				
Socialize	.164	.116	.210	.156
Regular drink	.332	.264	.267	.297
Night cap	.039	.124	.087	.077
Quiet drink	.334	.387	.344	.354
Other type	.202	.143	.159	.110
*Off-trade location*				
Own home	.822	.861	.807	.833
Someone else's home	.159	.109	.171	.144
Other off-trade location	.053	.044	.053	.050
*Activities while drinking (off-trade only)*				
Watching TV	.532	.559	.568	.548
Internet-related activity	.274	.240	.319	.271
Games and leisure	.236	.165	.276	.219
Chores	.256	.056	.085	.155
Get ready	.016	.016	.021	.017
N	16,839	12,340	6,779	35,958

*Notes*. RTD = ready-to-drink, eg cocktails.

**Table 2.** Summary statistics by food choice (meal consumed, snack only, no food) – on-trade occasions.

Discrete variables				
Food choice	Meal	No food	Snack	Mean
*Individual characteristics*				
Male	.528	.692	.572	.623
Female	.472	.308	.428	.377
Social class AB	.374	.315	.351	.339
Social class C1	.203	.214	.214	.210
Social class C2	.206	.208	.214	.208
Social class DE	.218	.263	.221	.243
Household income (<20k)	.22	.284	.262	.259
Household income (20k–35k)	.264	.277	.266	.272
Household income (35k–55k)	.251	.214	.231	.229
Household income (>55k)	.154	.136	.142	.143
Full-time worker	.493	.530	.559	.520
Other employment status	.507	.470	.441	.480
Children in the household binary	.275	.216	.267	.242
*Gender composition of the drinking occasion*				
Male alone	.025	.108	.052	.074
Female alone	.005	.014	.011	.011
Male pair	.048	.092	.077	.075
Female pair	.056	.026	.043	.038
Mixed pair	.286	.134	.166	.19
Male group	.071	.204	.133	.151
Female group	.059	.036	.053	.045
Mixed group	.508	.434	.537	.47
*Companionship while drinking*				
With family	.310	.1130	.211	.192
With friends	.411	.572	.570	.515
With partner	.409	.220	.274	.292
With other	.099	.107	.108	.104
*Mood of the occasion*				
Wind down	.106	.152	.167	.137
To have time for themselves	.276	.134	.153	.186
To bond	.217	.215	.250	.219
Have a laugh	.120	.194	.189	.168
*Duration of the occasion (h)*				
Duration (<1)	.087	.184	.115	.144
Duration (1–2)	.620	.495	.516	.541
Duration (>2)	.293	.321	.369	.315
*Alcohol consumption (units)*				
0–2	.142	.093	.104	.111
2–3	.288	.205	.217	.235
3–5	.134	.146	.141	.141
5–12	.269	.357	.309	.322
12–20	.094	.127	.139	.117
>20	.072	.073	.09	.074
*Main drinking beverage*				
RTD	.029	.026	.035	.028
Spirits	.155	.172	.177	.166
Cider	.138	.134	.156	.137
Wine	.258	.087	.135	.152
Beer	.421	.581	.497	.517
*Start time of the drinking occasion*				
Morning	.038	.041	.043	.04
Lunch	.220	.115	.122	.152
Afternoon	.240	.236	.219	.236
Evening	.481	.499	.560	.499
Night	.020	.109	.056	.073
*Day of the week*				
Weekdays	.425	.397	.382	.406
Weekend	.408	.458	.452	.44
Sunday	.167	.144	.166	.154
*On-trade venue*				
Modern bar	.143	.192	.201	.176
Traditional pub	.181	.411	.373	.327
Food pub	.323	.153	.168	.214
Restaurant	.309	.029	.077	.132
*Activities while drinking (on-trade only)*				
Watching TV	.084	.139	.185	.124
Game quiz	.047	.072	.107	.067
Pub quiz	.020	.030	.039	.028
Active game	.053	.089	.127	.080
Music game	.026	.051	.06	.043
Live music	.115	.168	.207	.153
Drink outside	.104	.126	.175	.123
Other on-trade activity	.114	.102	.133	.11
N	4,101	6,451	1,135	11,687

*Notes*. RTD = ready-to-drink, eg cocktails.

The data reveal the circumstances in which alcohol is consumed. Most available variables describe the characteristics of episodes rather than individuals. Consequently, these data are not susceptible to methodological individualist interpretation as it is less about individual persons and more about the pattern of behaviour by event type. The explanation is therefore not primarily in terms of personal capacities, resources, or decisions, rather more in terms of norms governing the arrangements of drinking episodes. We treat the event as the vector that results in the exposure of participants to certain styles and levels of alcohol consumption.

The main dependent variable for current purposes has three levels, drinking without food, with a meal, or with a snack. Alcovision defines “snack” simply as “light snack” when referring to domestic settings but specifies three sub-categories in on-trade locations: “crisps/nuts or other bagged snacks”, “sandwich/baguette/panini”, and “light snack/bar snack”. Note that the primary focus, militated by the data, is drinking events, so when discussing drinking with meals we are not making statements about all meals.

Measures of factors determining the character of events include the following: group size and composition; gender composition and the social relationships between participants; temporal parameters of time of day, day of the week, and duration; spatial parameters, whether at home or away, and if away at what type of venue and in what type of location (city centre, residential, rural, etc.). Sociodemographic characteristics of individual respondents include age, household income, employment status, class (market research social grades – AB, C1, C2, DE), gender, and urban/rural residence. While the survey also includes batteries of questions about mood, reasons, and venues, most of these do not discriminate between types of event, so we retain information about only a few which do, such as “winding down” or “having a laugh”.

The analysis compares the bivariate associations between the three types of event and the situational features of drinking occasions. The details for the models used to test for statistical differences between samples are explained in the Appendix (see footnote 11). Almost every measured difference is statistically significant because of the very large numbers of observations. However, for purposes of interpretation, we discuss only very strong associations. The focus of attention is the relative frequency of the interdependence of features of a social situation with the presence of food.

In accounting for the differences between types of event – with meals, snacks, or without food – the effects of situational features, where sufficiently strong, are discussed in turn: amount and type of alcohol consumed; time of day and day of the week; duration of the event; status of companions; mood and purpose of events; and concurrent activities. Differences by class and gender are then noted.

While Kantar data have been collected since 2001, methods of data collection and sampling have changed, radically in 2008. This paper concentrates on 2016. An examination of the data for 2009, the earliest comparable data point, shows no observable main trends.^iii^

## Findings

The data show that home was the principal location for drinking in 2016, on-trade occasions representing slightly less than one-quarter of all events reported. Occasions on- and off-trade are so differently structured that we describe them separately.

### Off-trade events, 2016

A majority (66%) of drinking events at home are accompanied by food, either a meal (47%) or a snack (19%).

More units of alcohol are consumed with food, on average 7.5 units with meals and 7.2 with snacks, compared with 5.9 at events without food. Quantity and type of beverage vary. Non-food events are more likely to involve less than 2 units of alcohol, meals more than 5. Wine is much more often present at meals, and beer and spirits at non-food events.

As Table 1 indicates, meal events are more likely to occur on Sundays, vestigial evidence of the British Sunday lunch accompanied now by alcohol, and drinking without food occurs less frequently on Sundays. “High” weekend times (defined as Friday evenings and Saturday), when Britons most frequently socialise both at home and away, mark the highest concentration of weekly domestic alcohol consumption but with little difference in the proportion of events with and without food. The weekend sees 43% of all events (42% meals, 44% non-food). Of the meals, 71% occur in the evening, as is typical in 21st century Britain for most domestic major meals, with the possible exception of Sundays (Yates and Warde, 2015).

When drinking occurs alongside a meal the event is more protracted, 24% last over 2 hours. Events without food take much less time, with 38% lasting less than 1 hour. Meals occur most predominantly in the evening, as do the other types of event, but drinking without food is more common after 10 pm (15% of non-food events). Meals are described predominantly as involving a regular or a quiet drink. Non-food occasions are more frequently described as a quiet drink or a nightcap.

The company present is an important parameter of the different types of event. A partner was present at 51% of meals, but at only 41% of non-food events. Family members were present at 29% of meals compared with 15% of non-food events. The implicit importance of evening household meals is corroborated by the gender composition of companions. Meals at home are much more likely to have either mixed pairs (couples/partners) or mixed groups present. (14% of meals have only a single person present.) Events without food are disproportionately likely to involve men, alone or in pairs.

Events of domestic hospitality are frequent; 14% of all events occur in the home of someone other than the respondent. Given the norm of reciprocity (Gouldner, 1960; Warde et al., 2020a), such an event will have a roughly even chance of being at the respondent's home. We might therefore infer from Table 1 that approximately 30% of all domestic events involve sociable mixing between households. Meals, and more especially snacks, are served on occasions when guests are present. A greater proportion of snack events have friends present than do meals. Offering small quantities of food, although not obligatory, is common when entertaining friends or non-resident kin.

Asked about activities occurring concurrently with drinking at home, respondents unsurprisingly reveal that drinking is not the only, and therefore perhaps not the primary, focus of attention. Over half of all occasions (55%) include watching television, more than one-quarter (27%) using the Internet, and more than one-fifth (22%) playing games. There is not much variation across the types of event, although snacks are more commonly associated with Internet usage and games. However, drinking episodes around meal events include chores in 26% of instances, 40% of which are explicitly cooking, capturing a habit, more common among women than men, of drinking during meal preparation.

Drinking at home with meals is more common in households with higher incomes and higher socioeconomic status. The poorest households are less likely to be present at meals where alcohol is consumed. The higher classes (AB) opt to drink alcohol with meals; meals have more members of the professional managerial classes present, while working classes are more likely to drink at home without a meal. A degree of class polarisation is apparent.

Differences between events reflect frequently observed gender differences (see Table A3). Men report greater involvement in each of the three types of event. Difference is greatest for non-food events, 64% of which were reported by men and only 36% by women. Despite women doing most of their drinking at home, men register 50% more of such events. On average, individual female respondents record 2.5 events during a 7-day period (whereas men report three events) comprising a greater proportion of meals and significantly fewer non-food events. Of events reported by men, 44% are meals, 19% snacks, and 37% non-food. For women, 52% of events are meals, 18% snacks, and 30% non-food. Men exceed women by 10%–20% in units of alcohol consumed.^iv^ Women do more of their drinking in the evening, with a greater proportion at meals. They define their experience less often as a regular drink than do men, perhaps because food is uppermost in consciousness. Wine was the primary drink at domestic meals for 55% of women but only 38% of men. The proportions for beer were 14% and 35%, respectively.

Men report 75% of meals as being in mixed company – with one or more companions – but eat alone a little more often than women. Women report fewer meal events during the week than men, but more at the weekend. By six percentage points their meals were more likely to be at someone else's home. Meals occur mostly in mixed company, but women are more likely than men to have family and friends present as well as their partners. The evidence about companions suggests that the meals reported by men are mostly “family meals”.

### On-trade events, 2016

On licensed premises, non-food events comprise 55% of all sessions and meals 35%. Meals outweigh snacks by 4:1. Average consumption of units of alcohol is greatest for snacks, least for meals. Of events in public places involving the consumption of alcohol, 62% are reported by men. Gender differences are more pronounced at on-trade events and require more explicit discussion when describing situational characteristics.

Regarding beverages, men consume beer much more often than women at all types of event and especially when no food is involved. In non-food settings, women drink more beer and spirits. Wine is the principal beverage for women in only 21% of events, although that is six times more than men. Men drink wine with meals more often than on other occasions, but wine remains four times less common than beer with meals. Non-food events involving women occur most often in pubs and modern bars. One-third of all meals reported by women are in restaurants, another third in “food pubs”, and most of the remainder in modern bars, traditional pubs, and family pubs.^v^ Men when eating a meal, by comparison, do so more often in traditional public houses than restaurants.

As Table 2 shows, meal events rarely take less than 1 hour. Non-food events are twice as likely to last less than 60 min (18% cf. 9%). Of meal events, 62% last between 1 and 2 hours, compared with 52% of non-food events. The proportion of meals occurring at the weekend is smaller than for non-food events, although on Sundays meals are more common. This echoes older social rhythms in Britain when Fridays and Saturdays were principal times for drinking away from home and Sundays were more sedate.

Compared with domestic events, on-trade non-food episodes of brief duration (less than 1 hour) are fewer and more last between 2 and 4 hours. Higher levels of alcohol are consumed, with beer more popular, and wine and spirits less frequently the primary beverage. Snack events are the type most likely to exceed a duration of 2 hours, implying that engagement with additional activities prolongs an episode and encourages recourse to convenient forms of minor nourishment.

Meal occasions provide greater opportunity for family and household members to drink in public than events comprising only drinking. Family members are in attendance at meals; a partner being present in 41% of cases, children in 17%, and other family in 31%. Friends were present at 41% of meals but 57% of non-food events, the latter featuring only 22% of partners, 4% of children, and 11% other family. Although in absolute terms men eat out more often, they report a much higher proportion of non-food events and a smaller proportion of meals than women. Men report 30% meals, 61% no food, and 9% snacks. The equivalent for women is 44%, 45%, and 11%, respectively. When they drink, women are more likely than men to be at events where food is served. Men meet in groups containing only other men at non-food events five times more frequently than women; of all occasions, 40% are men-only but only 8% women-only. At non-food events, men present are on average 14 years older than women, implying that ageing men are the customers in commercial establishments where no food is served. Male groups are more likely to come together at non-food events (30% cf. 12% of women), which are often described as bonding or having a laugh.

A high proportion of meal events (28%) are accounted for as “having time for myself, partner or family”, an indication that eating together away from home is an important part of the routines of many couples and partners. Non-food events, and also snacks, are more likely to cite “winding down” or “having a laugh” as a rationale. On-trade events often include complementary activities; live music (15% of events) was the most frequent, and watching TV and drinking outdoors each registered 12% of events. Complementary activities were least likely to occur alongside meals and most likely to involve snacks, suggesting that meals are a sufficient focus of attention in their own right while other entertainments and pastimes are accompanied by snacks.

Members of the professional and managerial classes (social grade AB) and households with higher incomes more often report eating meals while drinking. Events without food are more or less class neutral; working class people are equally likely to be present at non-food occasions. Meals attract more class AB men and proportionately fewer from class DE (see Appendix Table 4 which describes gender differences). Working class men are less likely to eat while drinking in both commercial and domestic settings. Working class women drink with meals less than their professional and managerial counterparts, but there is no class difference apparent at on-trade events.

The majority of public non-food events for women occur in mixed groups (59% of all such events, compared with 37% for men). Women are more likely to be accompanied by a partner at non-food occasions (33% cf. 17%). They also report family members present on 14% of occasions (cf. 10% for men). Thus, the conventions surrounding drinking in public for men and women remain differentiated. Women are less likely to drink or eat alone, and they more often have male companions. Only 0.5% of meals have a woman alone, compared to 2.5% with a man alone, confirming that women remain averse to eating out unaccompanied. In less than 5% of non-food events involving women, the woman is alone (cf. 16% men). Overall, women are more likely to eat when drinking, a tendency that increases monotonically with age. Conversely, the younger a woman the more she attends events without food. Age effects can also be observed on companions present. The youngest and the oldest age groups drink more frequently with friends, while the middle aged, who are more likely to have dependent children, are more often accompanied by partners or family.

## Discussion

### How food matters

The presence or absence of food marks significant differences in the social arrangements surrounding drinking. Meals, snacks, and drinks have different social meanings. The three types of events vary in terms of their procedures and purposes. They have different participants, companionship structures, and consumption patterns, and they deliver different experiences as a result of their different arrangements. The greatest contrast in form is between meal events and non-food events, with snack events mostly having intermediate characteristics. Most drinking events occur at home and a substantial proportion involve meals, a significant emergent facet of British alimentary and drinking culture. Demarcation between types of event is stronger in on-trade than in domestic settings.

A major determinant of the character of both meal and non-food occasions is whether they occur in domestic or commercial settings. Commercial non-food events attract a greater proportion of men and a smaller proportion of women, but there is little other sociodemographic variation by location. However, the gender composition of parties varies significantly; in public fewer men and women drink alone or in mixed pairs, male groups and mixed groups are much more prevalent, and there are fewer partners and many more friends. Unsurprisingly, sectors also differ in the complementary activities that occur alongside drinking. On-trade venues offer dancing, musical performances, quizzes, etc., which are very rarely part of domestic provision. However, at home, complementary activities occur in similar proportions at all three types of event. The overall impression is that on-trade events, whether meals or just drinks, are more convivial and less rushed, while domestic events are privatised, reserved for partners and other household members.^vi^

### Meals and snacks

Meals contrast most strongly with non-food events but are very similar irrespective of sectoral setting. For example, in both domestic and public settings, meals result in more friends being present, are more frequently daytime appointments, and last longer. Thus, the event type has some integrity; the same norms or constellations of arrangements persist whether home or away. This suggests that explanation of diversity in the character of drinking occasions lies primarily in the different understandings and conventions that define types of social occasion.

Meals are a minor element of commercial provision, notwithstanding the growing importance of catering for licensed businesses. Most on-trade events occur without food and only one-third (35%) involve a meal. Differences between meals at home and those in pubs and restaurants are limited. Meals away from home are more likely to involve friends and non-resident kin, but still partners and other household members comprise a high proportion of companions. Duration, units of alcohol consumed, time of day, and beverage preference are similar.

Meals are more common in domestic settings. Perhaps unsurprisingly, given the preponderance of households whose members are likely to be together at home in the evening and at the time when most eat dinner, variation by gender is not very great. Partners eat together both at home and on commercial premises but are less frequently co-present when just drinking. The trend towards most people eating their main meal at home in the evening, a convenient arrangement for an increasing proportion of dual-earner households, offered new opportunities for drinking (Vogler, 2020). Most drinking of alcohol since the late-Victorian era has occurred primarily in the evening and it was maybe a small step to incorporate drinking into a major meal occurring at the end of the working day. Domestic meal formats have adjusted to incorporate alcohol as off-trade sales rose.

The legitimacy of drinking alcohol with meals increases opportunities to drink for both men and women. Meals in restaurants for the sake of entertainment, where drinking is central to the format, only became a common practice for the majority of the population in the 1980s (Burnett, 2004). Drinking alcohol at home with the main meal of the day, previously the scene for only a small minority of the population has also become common within living memory (see Burnett, 1999; cf Warren, 1958). Now, 45% of domestic drinking events for Britons who ever drink alcohol are accompanied by a meal. Factors contributing to this shift will include the following: learning from eating in restaurants; foreign holidays; migration from countries where drinking with dinner is common; supermarket sales of drink as part of a food shopping excursion; relative decline in price in the context of rising incomes; relaxation of negative attitudes towards alcohol; more women drinking; a larger professional and managerial class inheriting elite practices including dinner parties; greater likelihood of using alcohol for ritual occasions of celebration; and the promotion of alcohol by the drinks industry in the absence of discouragement by a state that obtains significant revenue from taxation. Among these, one might hypothesise that drinking with meals was learned in the late 20th century from behaviour in the commercial sector and was subsequently incorporated into domestic routines.

Our findings suggest a gradual shift, accelerating since the millennium, towards drinking with meals, especially at home. The dinner table is now the site of a substantial proportion of all drinking events. This additional setting for legitimate alcohol consumption has emerged to provide the opportunity for regular and routinised events involving moderate levels of drinking at home. The reasons adduced for domestic events – like “winding down”, “just a quiet drink”, “a regular drink” – suggest a degree of normalisation, typical of mundane social practices (Warde, 2016).

Drinking with dinner remains more common among the professional classes and is the occasion when wine is most prevalent. The presence of alcohol probably elevates the meal in a hierarchy of occasions; more important meals justify wine and beer, although the normalisation of drinking on weekday evenings may progressively flatten any effect. The relative popularity of wine with meals, both at home and away, is consistent with trends towards greater diversity and flexibility in consumption patterns. An extended period of informalisation in manners and etiquette, across many fields of cultural behaviour and lifestyle, weakens previously observed rules of behaviour (Wouters, 2008). For example, older etiquette prescribed drinking red wine with cheese, but now sweet, sparkling, and white wines are recommended for reasons of innovation, fashion, and gastronomic principle. Personal preferences for particular beverages have become admissible justification for unorthodox pairings of food and drink.

Informalisation may be reflected in changing class norms. Men in class AB drink with meals, whereas those in Class DE still do not. However, other indications of class differentiation are weak and perhaps diminishing. There is no social compulsion to mark position in a status hierarchy by selection of beverage, especially among women. Symbolic aspects of drink selection persist nevertheless. Intermediaries advise that beer should be drunk with South Asian dishes, and water has undergone a major revival, with sparkling water in restaurants a signifier of cultural capital and fashion (Warde et al., 2020b).

The social regulation of meals is well documented, with its consequent predictable variations in format and style. A hierarchy of meal events is fairly securely established, expressed through quantity of food, type of food, degree of elaboration, definition of the occasion, duration, company, location, and calendric significance (e.g., feast days) (e.g., Douglas, 1972; Warde, 2016). It is more difficult to make an equivalent argument for drinking occasions. A latent class analysis of events conducted by Ally et al. (2016) indicated meaningful differentiation of types of occasion, but without suggesting any definitive hierarchical structure. Events may be described along several dimensions – heavy or light sessions, big nights out, quick ones at the local, routine or unexpected, memorable or casual, weekend or weekday. Perhaps “the big night out” stands at one pole, a quiet drink alone at the local pub at the other. Perhaps also on-trade events are more prestigious or more meaningful occasions for drinking. Possibly the events most concentrated around the processes and pleasures of drinking – or at least the most protracted and convivial – are those accompanied by snacks. That is to say, meals out are evaluated as eating occasions rather than drinking opportunities. They are highly appreciated and possibly have most social kudos. However, arguably, the meal is the main point of the activity, with the drinks a subsidiary consideration. That seems less so with the snack.

The snack is an ill-defined and often residual alimentary category (Warde & Yates, 2017), although it is perhaps less ambiguous in the context of drinking. Taking snacks with drinks is not markedly gendered, although women do report more snack events on-trade than do men. They last longer, attract people of the highest socioeconomic status (class AB), and involve more family members as companions compared with non-food events. Participants in snack events have a lower mean age and friends are more likely to be present.

The snack signifies conviviality, facilitates a greater degree of sharing, and gives beverages a higher profile or standing. At events of domestic hospitality, offering a guest at least a small quantity of food to accompany an alcoholic drink is almost obligatory but is less likely to be a meal than a snack (Douglas, 1972). Snacks promote sociability beyond the family and also signify informality. A snack, while requiring less effort and bearing less symbolic significance than a meal, still signifies the importance of providing food alongside drink when offering hospitality to friends or non-resident kin. Snacks appear more commonly at on-trade events of longer duration, in the evening, in mixed company, and at live events. Part of the explanation is that more prolonged events away from home probably require some sort of solid refreshment to maintain bodily comfort and counteract the effects of alcohol – an emergency ration to sustain lengthy drinking sessions. In addition, when complementary activities occur more snacks are reported. The issue deserves further research.

### Gender differentiation and contemporary practice

Drinking without food remains the principal form of on-trade consumption. It is more popular with men, and groups much more often comprise only men. Such events ground the stereotype of traditional, masculine drinking involving consumption of rather a lot of beer in British pubs. While an iconic representation of British pub culture, men drinking together constitute less than one-fifth (17%) of on-trade events and therefore merely 4% of all drinking events. Women drinking without food use a wider variety of outlets and are much more likely to drink in mixed company. At home, while men still drink more often than women, gender differences are less stark with men and women alike describing non-food occasions most often as a quiet drink or a nightcap.

The conventions governing drinking at home are fairly similar for men and women. No qualities of an event are exclusive to one or other sex. Men and women participate in every type of event, although at different rates. Thus while Brierley-Jones et al. (2014) using focus groups could identify two basic ideal typical formats for drinking episodes, coded masculine and feminine and recognisable as stereotypes, they are not prominent in the aggregate behaviour of the population as a whole. The heterogeneous array of available formats is shared, although not exactly equally, by men and women alike. Men drink more heavily, more frequently, more beer, and more often without food both home and away. Women are more likely to eat something with their drinks and remain more averse to eating out or drinking unaccompanied. Nevertheless, when engaged in a specific type of event, men and women behave in much the same ways, offer the same reasons for participating and justifications for the arrangements – which is evidence of a practice shared. Women's consumption pattern is as much the consequence of types of event attended as of any intrinsic inclination or disposition. Fewer women will be at the bar, and they will more likely be in the company of family members, while the dining room will contain proportionately more women, as well as more members of the professional and managerial classes and more family groups. Alcoholic beverages have their times and places, now very often in association with food. Variation in performances is best explained by the conjunction of arrangements governing different settings and the imperatives of other practices like employment and household responsibilities, different household size and type, and the temporal rhythms of the week (Grignon, 1993; Southerton, 2020).

Settings are important for understanding eating-and-drinking. Adjusting flexibly to settings demonstrates personal and procedural competence to perform in accordance with the norms and conventions of established practices. The quiet drink, the winding down period, and the regular drink primarily with meals are the most common re-descriptions of all kinds of event. Big nights out, with long periods of sustained drinking, comprise a small proportion of all occasions, suggesting that the focus of research on episodes of excessive intoxication hampers appreciation of the more common and normal ways of using alcoholic beverages (see Thurnell-Read, 2020). In many episodes, drinking is subsidiary to other activities, most notably in relation to drinking with meals. Complementary activities are prevalent both at home (54%) and away (49%). The majority of episodes involve intake of 5 or fewer units of alcohol. Hence one might view most drinking events as rather innocuous. Occurring only occasionally, most involve relatively modest amounts of alcohol consumed over a period of 1 or 2 hours. Domestic events have lower levels of alcohol. Even at on-trade events, people's companions are very often partners and family members. Most drinking is fairly mundane. Nevertheless, while any single occasion may be mundane, repetitions might be a problem from the perspective of public health. Whichever, it may be productive to address behaviour change in different ways depending on where alcohol sits within the occasion and in sequences of occasions.

## Conclusion

This paper points to theoretical and methodological complications arising when more than one practice is relevant to the understanding of patterns of behaviour. People doing more than one thing at a time is inconvenient for analyses concentrated on a single focal practice, as recent discussions of practice theory reveal (Blue et al., 2021; Hennell et al., 2020; Warde, 2013). The paucity of literature concerning drinking with food is a consequence; food studies mostly neglect to inquire about drinks and alcohol studies tend to ignore eating. This paper reunites these activities by presenting a novel preliminary analysis of the co-occurrence of types of eating event and episodes of alcohol consumption. Employing a dataset well suited to the understanding of behaviour in specific situations, we have estimated the degrees to which what was drunk and eaten in 2016 varied systematically depending upon the number and status of companions, time of day, day of the week, spatial location, and so on. That the results were little different in 2009 suggests that stable norms and conventions guide the practices.

Much drinking occurs simultaneously with subsistence and recreational activities, within and outside the home. Knowing whether drinking or eating are primary or subsidiary activities at an event is critical for understanding behaviour, not least because of its potential consequences for health-related interventions (Meier et al., 2017). People adjust their drinking behaviour with reference to eating, and *vice versa*, which potentially modifies the fundamental principles and procedures of both eating and drinking practices.

To our knowledge, no comparable surveys have been analysed to address this question, hence it is uncertain whether the same associations exist in other national contexts. Future research might usefully pay greater attention to the coincidence of eating and drinking, not least because the social structuring of different types of event affects levels of alcohol consumption, a matter important in its own right and useful when devising interventions to change behaviour. Collecting more elaborate and specifically targeted data on types of occasion and sociodemographic characteristics of individuals would pay dividends. A purpose-designed survey to explore the intersection of eating and drinking would be ideal because the Kantar survey is not of the whole population. Rather it records only the eating-and-drinking behaviour of a section of the population who imbibed at least 1 unit of alcohol in the previous 7 days. Consequently, the data collected are asymmetrical, a record of what those who drink eat, not what those who eat drink. Comparative studies, perhaps contrasting societies with different ratios of eating and drinking in domestic and public settings, would also enhance understanding of the association.

## Supplemental Material

sj-docx-1-nad-10.1177_14550725231157222 - Supplemental material for Situated drinking: The association between eating and alcohol consumption in Great Britain Click here for additional data file. Supplemental material, sj-docx-1-nad-10.1177_14550725231157222 for Situated drinking: The association between eating and alcohol consumption in Great Britain by Alan Warde, Alessandro Sasso, John Holmes, Monica Hernández Alava, Abigail K. Stevely and Petra S. Meier in Nordic Studies on Alcohol and Drugs

## Notes

1. Significantly, there is no term in common use to depict the integration of the two activities. The only precise term is “victuals”, which sounds rather archaic.

2. Considering only the section of the population who drink at home at least once a week, Britain was top of the list.

3. Tests show differences between the 2 years to be small, even if statistically significant due to the large sample size.

4. Men report units of alcohol as 8.09 at meals, 6.22 non-food, and 7.51 snacks, while women claim 6.79 at meals, 5.31 non-food, and 6.76 snacks.

5. The denotation in the survey of venues is neither precise nor sophisticated but is still informative.

6. Meals at home are described as a “regular drink” more often with meals than at the other two types of event.
