# Supplementary material for: Situated drinking: The association between eating and alcohol consumption in Great Britain
Source: Nordisk Alkohol Nark. 2023 Feb 21;40(3):301–18. doi: 10.1177/14550725231157222 (PMC10225964; doi:10.1177/14550725231157222)
Supplement: sj-docx-1-nad-10.1177_14550725231157222 - Supplemental material for Situated drinking: The association between eating and alcohol consumption in Great Britain [file sj-docx-1-nad-10.1177_14550725231157222.docx]

Appendix online: **Situated drinking: the association between eating and alcohol consumption in Great Britain**

***Data quality and technicalities***

Kantar Alcovision is collected with the method of quota sampling used to draw a representative sample of the adult population of Great Britain (excluding Northern Ireland) in terms of the following socio-demographic characteristics: age, gender, region and social grade. In addition, survey invitations are split evenly within each month of the year to ensure that every day of the year is covered. The timespan of Kantar Alcovision is from 2009 onwards. However, from 2017 the sample size has been reduced possibly reflecting a cut in financial resources for data collection by Kantar. Hence, the present analysis focuses on 2016 (the most recent data with the largest sample size) and compares the results with 2009 to investigate changes over time.

Kantar Alcovision has a complex multilevel data structure capturing information at three levels: drink-level information, describing the characteristics of drinks reported by the respondents (e.g. type of drink size, and strength) within each drinking occasion of the week.^[[1]](#endnote-1)^ Occasion-level information, describing the characteristics of the reported drinking occasions such as location, starting time, type of venue, etc. Individual-level information, referring to personal characteristics of the respondent that do not vary between drinking occasions (e.g. gender, socio-economic class, household composition, etc.). The focus of this study is on occasion and individual characteristics. The full list of variables included in this analysis is described below.

Regarding personal characteristics, we analyse the following variables of interest: age, gender, the presence of children in the household, full-time working status, social grade, and household income. Age is a continuous variable ranging from 18 to 90 years old. Gender is a binary variable capturing whether the individual is male (or female otherwise). Social Class is described by a set of mutually exclusive binary variables (namely AB, C1, C2, DE) reflecting the standard market research classification of social grades, where AB is the highest socio-economic group and DE is the lowest. Household income is included using the following set of mutually exclusive binary variables: less than £20,000; £20,000-34,999; £35,000-55,999; over £55,000. Full-time worker is a binary variable describing whether the individual is employed on a full-time contract. ‘Children in the household’ is a binary variable indicating whether the respondent has any dependent children living in the household.

Regarding occasion characteristics, we focus on the following variables of interests: gender composition of the drinking occasion, companionship while drinking, mood of the drinking occasion, duration of the drinking occasion, average alcohol consumption per occasion, main type of beverage, start time of the drinking occasion, day of the week, type of occasion, off-trade location, and activities while drinking.

‘Gender composition of the drinking occasion’ describes the gender of all the individuals taking part to a drinking occasion. This is captured with the following set of dummy variables: male alone, female alone, male pair, female pair, mixed pair, male group, female group, mixed group.

Drinking companionship is a set of dummy variables describing the type of company (relatives, friends, partner, or other individuals including work colleagues) when the respondent is not drinking alone. Drinking companionship is captured with a multiple-choice question.

Variables describing the drinking location and the activities undertaken while drinking are specific to the type of sector (either off-trade or on-trade). For example, the set of binary variables describing the on-trade venues are club, modern bar, traditional pub, family pub, food pub, student pub, social club, restaurant, or other on-trade venue. The variables describing off-trade locations are own home, someone else’s home, and other off-trade venue.

Two multiple-choice questions are used in the survey to capture a long list of potential on-trade and off-trade activities (33 in total) undertaken while drinking. We collapse these categories into a shorter number of variables based on their similarity. Specifically, the variables describing on-trade activities are: watching TV, game quiz (also including ‘games machine’ and ‘fruit machine’), pub quiz, active game (also including pool, darts and bowling), music game (including taking part or watch karaoke, or play juke box), live music, drink outside, or a category describing other activities. The off-trade activities are watching TV, using internet (including ‘play games console’, ‘browse the internet’, ‘gamble online’, ‘social network online’), play games (including ‘play cards (not online)’, ‘play games/board games’, ‘read’, ‘listen to music’, and ‘gardening/hobbies’), doing the chores (including ‘cooking’, ‘shop online’, ‘work study’, and ‘housework’), and getting ready.

A survey question is used to elicit the mood/reason of a drinking occasion. Given that this variable has some categories with very low responses, we collapse some of these into a smaller number of categories. Hence, the final set of dummy variables describing the reason for drinking are the following: ‘wind down’ (also including the ‘chill out’ response to the questionnaire); ‘to have time for themselves’ (also includes ‘to have time for my partner or relative’); ‘to treat/reward myself’; ‘to refresh, recharge or invigorate’; ‘to bond/be sociable’; ‘to let go’ (also including ‘up for it’ or ’to get drunk’); ‘having a laugh’ (also including the response ‘getting in the mood’).

Variables describing gender composition, drinking companionship, reason for the occasion, and drinking venue are not mutually exclusive as they may vary within the same drinking occasion. For example, the gender composition of a drinking occasion could be a combination of 'drinking alone’ and ‘mixed pair’ if an individual starts pre-drinking alone and then his/her partner arrives. In contrast, the remaining set of variables are mutually exclusive which means that they indicate characteristics that do not vary within the drinking occasion. For example, we use three binary variables to indicate whether the total duration of a drinking occasion (namely less than one hour, between one and two hours, or over two hours). Units of alcohol consumed per occasion is a continuous variable ranging from 18 to 40 units (40 is the maximum cap hence representing a consumption of 40 or more units). In addition, we generate a set of binary variables (0-2 units, 2-3 units, 3-5 units, 5-12 units, 12-20 units, > 20 units) as we are interested in capturing the proportion of occasions with specific consumption levels rather than focusing only on its mean. ‘Day of the week’ is a set of three binary variables capturing whether the occasion started on a weekday (before Friday 5pm), weekend (between 5pm on Friday until midnight on Saturday), or Sunday.

In this study, we present weighted summary statistics to investigate how individual and occasion characteristics differ across three types of drinking occasions: occasions with meal, occasions without any food, and occasions with snacks only.^[[2]](#endnote-2)^ In addition, we compare the results by trade sector and gender.^[[3]](#endnote-3)^ The year considered for this analysis is 2016. As we explained, we also compare the results between 2009 and 2016 to analyse potential changes over time. However, since we did not find any significant changes between these two periods, we present only the results for 2016. The results we present are by type of sector. As explained, we also examined results for men and women separately and test for gender differences which are reported in Tables A3 and A4.

**Table A1 - Summary statistics by food choice (meal consumed, snack only, no food). Off-trade occasions. Continuous variables**

|  | **N** | **Mean** | **SD** | **Min** | **Max** |
| --- | --- | --- | --- | --- | --- |
| **Food choice: meal** | | | | | |
| age | 16,839 | 44.429 | 17.309 | 18 | 87 |
| units | 16,839 | 7.504 | 7.5 | .13 | 40 |

**Food choice: no food**

| age | 12,340 | 44.533 | 16.136 | 18 | 90 |
| --- | --- | --- | --- | --- | --- |
| units | 12,340 | 5.899 | 6.046 | .13 | 40 |

**Food choice: snack**

| age | 6,779 | 43.217 | 16.159 | 18 | 87 |
| --- | --- | --- | --- | --- | --- |
| units | 6,779 | 7.21 | 7.287 | .13 | 40 |

**Food choice: all**

| age | 35,958 | 44.236 | 16.707 | 18 | 90 |
| --- | --- | --- | --- | --- | --- |
| units | 35,958 | 6.898 | 7.031 | .13 | 40 |

**Discrete variables**

| **Food choice** | **Meal** | **No food** | **Snack** | **All** |
| --- | --- | --- | --- | --- |
| Individual characteristics |  |  |  |  |
| Male | .550 | .643 | .601 | .592 |
| Female | .450 | .357 | .399 | .408 |
| Social Class AB | .369 | .294 | .332 | .336 |
| Social Class C1 | .206 | .207 | .189 | .203 |
| Social Class C2 | .198 | .219 | .203 | .206 |
| Social Class DE | .228 | .280 | .276 | .255 |
| Household Income (< 20k) | .229 | .290 | .279 | .259 |
| Household Income (20k - 35k) | .273 | .280 | .284 | .278 |
| Household Income (20k - 55k) | .253 | .214 | .200 | .229 |
| Household Income (> 55k) | .137 | .122 | .145 | .133 |
| Full-time worker | .454 | .479 | .478 | .467 |
| Other employment status | .546 | .521 | .522 | .533 |
| Children in the household | .261 | .298 | .335 | .288 |
| Gender composition of the drinking occasion |  |  |  |  |
| Male alone | .085 | .201 | .156 | .138 |
| Female alone | .055 | .085 | .076 | .069 |
| Male pair | .047 | .085 | .058 | .062 |
| Female pair | .034 | .038 | .039 | .036 |
| Mixed pair | .393 | .326 | .320 | .356 |
| Male group | .024 | .032 | .033 | .029 |
| Female group | .019 | .018 | .026 | .02 |
| Mixed group | .374 | .233 | .336 | .318 |
| Companionship while drinking |  |  |  |  |
| With family | .285 | .147 | .191 | .220 |
| With friends | .190 | .154 | .253 | .190 |
| With partner | .514 | .409 | .444 | .465 |
| With other | .025 | .019 | .027 | .023 |
| Mood of the occasion |  |  |  |  |
| Wind down | .348 | .401 | .340 | .365 |
| To have time for themselves | .189 | .166 | .174 | .178 |
| To treat/reward themselves | .088 | .093 | .083 | .089 |
| To have a break | .042 | .055 | .061 | .049 |
| To refresh, recharge or invigorate | .088 | .095 | .106 | .093 |
| To bond | .068 | .041 | .086 | .063 |
| To let go | .033 | .043 | .046 | .039 |
| Have a laugh | .055 | .050 | .100 | .061 |
| Other mood | .161 | .069 | .066 | .113 |
| Duration of the occasion |  |  |  |  |
| Duration (<1 hour) | .305 | .378 | .289 | .327 |
| Duration (1-2 hours) | .438 | .471 | .469 | .455 |
| Duration (>2 hours) | .237 | .151 | .242 | .218 |
|  |  |  |  |  |
| **Table A1 (continued). Discrete variables** |  |  |  |  |
| **Food choice** | **Meal** | **No food** | **Snack** | **All** |
| Alcohol consumption |  |  |  |  |
| 0-2 units | .144 | .237 | .197 | .186 |
| 2-3 units | .223 | .209 | .193 | .212 |
| 3-5 units | .133 | .156 | .134 | .141 |
| 5-12 units | .330 | .290 | .310 | .313 |
| 12-20 units | .097 | .071 | .100 | .089 |
| > 20 units | .073 | .037 | .066 | .059 |
| Main drinking beverage |  |  |  |  |
| RTD | .016 | .018 | .020 | .018 |
| Spirits | .155 | .263 | .242 | .209 |
| Cider | .114 | .126 | .150 | .125 |
| Wine | .458 | .230 | .256 | .342 |
| Beer | .256 | .363 | .332 | .307 |
| Start time of the drinking occasion |  |  |  |  |
| Morning | .012 | .027 | .022 | .019 |
| Lunch | .063 | .037 | .062 | .054 |
| Afternoon | .184 | .129 | .144 | .158 |
| Evening | .713 | .655 | .664 | .684 |
| Night time | .028 | .152 | .108 | .086 |
| Day of the week |  |  |  |  |
| Weekdays | .385 | .418 | .382 | .396 |
| Weekend | .422 | .438 | .458 | .434 |
| Sunday | .193 | .144 | .160 | .170 |
| Type of the occasion (off-trade) |  |  |  |  |
| Socialize | .164 | .116 | .210 | .156 |
| Regular drink | .332 | .264 | .267 | .297 |
| Night cap | .039 | .124 | .087 | .077 |
| Quiet drink | .334 | .387 | .344 | .354 |
| Other type | .202 | .143 | .159 | .110 |
| Off-trade location |  |  |  |  |
| Own home | .822 | .861 | .807 | .833 |
| Someone else’s home | .159 | .109 | .171 | .144 |
| Other off-trade location | .053 | .044 | .053 | .050 |
| Activities while drinking (off-trade only) |  |  |  |  |
| Watching TV | .532 | .559 | .568 | .548 |
| Internet-related activity | .274 | .240 | .319 | .271 |
| Games and leisure | .236 | .165 | .276 | .219 |
| Chores | .256 | .056 | .085 | .155 |
| Get ready | .016 | .016 | .021 | .017 |
| N | 16,839 | 12,340 | 6,779 | 35,958 |

**Table A2 - Summary statistics by food choice (meal consumed, snack only, no food). On-trade occasions. Continuous variables**

|  | **N** | **Mean** | **SD** | **Min** | **Max** |
| --- | --- | --- | --- | --- | --- |

**Food choice: meal**

| age | 4,101 | 40.319 | 17.074 | 18 | 90 |
| --- | --- | --- | --- | --- | --- |
| units | 4,101 | 7.174 | 7.62 | .03 | 40 |

**Food choice: no food**

| age | 6,451 | 41.976 | 17.104 | 18 | 87 |
| --- | --- | --- | --- | --- | --- |
| units | 6,451 | 8.178 | 7.34 | .16 | 40 |

**Food choice: snack**

| age | 1,135 | 38.27 | 16.03 | 18 | 85 |
| --- | --- | --- | --- | --- | --- |
| units | 1,135 | 8.567 | 8.249 | .16 | 40 |

**Food choice: all**

| age | 11,687 | 41.035 | 17.032 | 18 | 90 |
| --- | --- | --- | --- | --- | --- |
| units | 11,687 | 7.864 | 7.549 | .03 | 40 |

**Discrete variables**

| **Food choice** | **Meal** | **No food** | **Snack** | **All** |
| --- | --- | --- | --- | --- |
| Individual characteristics |  |  |  |  |
| Male | .528 | .692 | .572 | .623 |
| Female | .472 | .308 | .428 | .377 |
| Social Class AB | .374 | .315 | .351 | .339 |
| Social Class C1 | .203 | .214 | .214 | .210 |
| Social Class C2 | .206 | .208 | .214 | .208 |
| Social Class DE | .218 | .263 | .221 | .243 |
| Household Income (< 20k) | .22 | .284 | .262 | .259 |
| Household Income (20k - 35k) | .264 | .277 | .266 | .272 |
| Household Income (35k - 55k) | .251 | .214 | .231 | .229 |
| Household Income (> 55k) | .154 | .136 | .142 | .143 |
| Full-time worker | .493 | .530 | .559 | .520 |
| Other employment status | .507 | .470 | .441 | .480 |
| Children in the household binary | .275 | .216 | .267 | .242 |
| Gender composition of the drinking occasion |  |  |  |  |
| Male alone | .025 | .108 | .052 | .074 |
| Female alone | .005 | .014 | .011 | .011 |
| Male pair | .048 | .092 | .077 | .075 |
| Female pair | .056 | .026 | .043 | .038 |
| Mixed pair | .286 | .134 | .166 | .19 |
| Male group | .071 | .204 | .133 | .151 |
| Female group | .059 | .036 | .053 | .045 |
| Mixed group | .508 | .434 | .537 | .47 |
| Companionship while drinking |  |  |  |  |
| With family | .310 | .1130 | .211 | .192 |
| With friends | .411 | .572 | .570 | .515 |
| With partner | .409 | .220 | .274 | .292 |
| With other | .099 | .107 | .108 | .104 |
| Mood of the occasion |  |  |  |  |
| Wind down | .106 | .152 | .167 | .137 |
| To have time for themselves | .276 | .134 | .153 | .186 |
| To treat/reward themselves | .076 | .052 | .042 | .059 |
| To have a break | .084 | .099 | .087 | .093 |
| To refresh, recharge or invigorate | .071 | .101 | .094 | .090 |
| To bond | .217 | .215 | .250 | .219 |
| To let go | .029 | .052 | .048 | .044 |
| Have a laugh | .120 | .194 | .189 | .168 |
| Other mood | .104 | .072 | .058 | .082 |
| Duration of the occasion |  |  |  |  |
| Duration (<1 hour) | .087 | .184 | .115 | .144 |
| Duration (1-2 hours) | .620 | .495 | .516 | .541 |
| Duration (>2 hours) | .293 | .321 | .369 | .315 |
|  |  |  |  |  |
| **Table A2 (continued). Discrete variables** |  |  |  |  |
| **Food choice** | **Meal** | **No food** | **Snack** | **All** |
| Alcohol consumption |  |  |  |  |
| 0-2 units | .142 | .093 | .104 | .111 |
| 2-3 units | .288 | .205 | .217 | .235 |
| 3-5 units | .134 | .146 | .141 | .141 |
| 5-12 units | .269 | .357 | .309 | .322 |
| 12-20 units | .094 | .127 | .139 | .117 |
| > 20 units | .072 | .073 | .09 | .074 |
| Main drinking beverage |  |  |  |  |
| RTD | .029 | .026 | .035 | .028 |
| Spirits | .155 | .172 | .177 | .166 |
| Cider | .138 | .134 | .156 | .137 |
| Wine | .258 | .087 | .135 | .152 |
| Beer | .421 | .581 | .497 | .517 |
| Start time of the drinking occasion |  |  |  |  |
| Morning | .038 | .041 | .043 | .04 |
| Lunch | .220 | .115 | .122 | .152 |
| Afternoon | .240 | .236 | .219 | .236 |
| Evening | .481 | .499 | .560 | .499 |
| Night time | .020 | .109 | .056 | .073 |
| Day of the week |  |  |  |  |
| Weekdays | .425 | .397 | .382 | .406 |
| Weekend | .408 | .458 | .452 | .44 |
| Sunday | .167 | .144 | .166 | .154 |
| On-trade venue |  |  |  |  |
| Club | .026 | .068 | .062 | .052 |
| Modern bar | .143 | .192 | .201 | .176 |
| Traditional pub | .181 | .411 | .373 | .327 |
| Family pub | .105 | .074 | .093 | .087 |
| Food pub | .323 | .153 | .168 | .214 |
| Student pub | .331 | .163 | .168 | .223 |
| Social club | .012 | .013 | .014 | .012 |
| Restaurant | .309 | .029 | .077 | .132 |
| Other on-trade venue | .128 | .111 | .155 | .121 |
| Activities while drinking (on-trade only) |  |  |  |  |
| Watching TV | .084 | .139 | .185 | .124 |
| Game quiz | .047 | .072 | .107 | .067 |
| Pub quiz | .020 | .030 | .039 | .028 |
| Active game | .053 | .089 | .127 | .080 |
| Music game | .026 | .051 | .06 | .043 |
| Live music | .115 | .168 | .207 | .153 |
| Drink outside | .104 | .126 | .175 | .123 |
| Other on-trade activity | .114 | .102 | .133 | .11 |
| N | 4,101 | 6,451 | 1,135 | 11,687 |

**Table A3 - Summary statistics by food choice (meal consumed, snack only, no food) and gender. Off-trade occasions. Continuous variables**

| **Sample** | **Females** | | | | | **Males** | | | | |
| --- | --- | --- | --- | --- | --- | --- | --- | --- | --- | --- |
|  | **N** | **Mean** | **SD** | **Min** | **Max** | **N** | **Mean** | **SD** | **Min** | **Max** |
| **Food choice: meal** |  |  |  |  |  |  |  |  |  |  |
| age | 7,570 | 50.86 | 16.53 | 18 | 85 | 9,269 | 50.61 | 17.12 | 18 | 87 |
| units | 7,570 | 6.16 | 5.88 | 0.15 | 4 | 9,269 | 7.87 | 7.78 | 0.13 | 40 |
| **Food choice: no food** |  |  |  |  |  |  |  |  |  |  |
| age | 4,406 | 50.13 | 16.46 | 18 | 87 | 7,934 | 49.09 | 15.90 | 18 | 90 |
| units | 4,406 | 4.82 | 5.07 | 0.13 | 40 | 7,934 | 6.11 | 5.99 | 0.15 | 40 |
| **Food choice: snack** |  |  |  |  |  |  |  |  |  |  |
| age | 2,708 | 50.11 | 17.07 | 18 | 85 | 4,071 | 48.14 | 16.05 | 18 | 87 |
| units | 2,708 | 6.09 | 6.24 | 0.15 | 40 | 4,071 | 7.26 | 7.23 | 0.13 | 40 |
| **Food choice: all** |  |  |  |  |  |  |  |  |  |  |
| age | 14,684 | 50.51 | 16.61 | 18 | 87 | 7,280 | 47.03 | 17.11 | 18 | 90 |
| units | 14,684 | 5.75 | 5.75 | 0.13 | 40 | 7,280 | 8.41 | 7.57 | 0.03 | 40 |

| **Discrete variables** |  | | | |  | | | |
| --- | --- | --- | --- | --- | --- | --- | --- | --- |
| **Sample** | **Females** | | | | **Males** | | | |
| **Food choice** | **Meal** | **No food** | **Snack** | **All** | **Meal** | **No food** | **Snack** | **All** |
| Individual characteristics |  |  |  |  |  |  |  |  |
| Social Class AB | 0.271 | 0.186 | 0.218 | 0.236 | 0.338 | 0.262 | 0.317 | 0.306 |
| Social Class C1 | 0.307 | 0.299 | 0.320 | 0.307 | 0.309 | 0.315 | 0.282 | 0.306 |
| Social Class C2 | 0.228 | 0.224 | 0.204 | 0.223 | 0.196 | 0.233 | 0.211 | 0.212 |
| Social Class DE | 0.194 | 0.290 | 0.258 | 0.234 | 0.157 | 0.190 | 0.190 | 0.175 |
| Household Income (< 20k) | 0.253 | 0.330 | 0.337 | 0.291 | 0.202 | 0.264 | 0.237 | 0.231 |
| Household Income (20k - 35k) | 0.317 | 0.298 | 0.311 | 0.310 | 0.291 | 0.299 | 0.306 | 0.297 |
| Household Income (35k - 55k) | 0.208 | 0.167 | 0.169 | 0.189 | 0.280 | 0.234 | 0.203 | 0.249 |
| Household Income (> 55k) | 0.106 | 0.078 | 0.064 | 0.090 | 0.144 | 0.127 | 0.179 | 0.144 |
| Full-time worker | 0.296 | 0.286 | 0.283 | 0.291 | 0.510 | 0.546 | 0.564 | 0.533 |
| Other employment status | 0.704 | 0.714 | 0.717 | 0.709 | 0.490 | 0.454 | 0.436 | 0.467 |
| Married | 0.352 | 0.362 | 0.376 | 0.359 | 0.320 | 0.369 | 0.356 | 0.345 |
| Children in the household binary | 0.222 | 0.262 | 0.253 | 0.240 | 0.214 | 0.248 | 0.312 | 0.244 |
| Gender composition of the drinking occasion | | | | |  |  |  |  |
| Male alone | 0.000 | 0.000 | 0.000 | 0.000 | 0.155 | 0.312 | 0.261 | 0.233 |
| Female alone | 0.160 | 0.267 | 0.223 | 0.203 | 0.000 | 0.000 | 0.000 | 0.000 |
| Male pair | 0.000 | 0.000 | 0.000 | 0.000 | 0.090 | 0.132 | 0.096 | 0.107 |
| Female pair | 0.068 | 0.091 | 0.092 | 0.079 | 0.000 | 0.000 | 0.000 | 0.000 |
| Mixed pair | 0.452 | 0.408 | 0.379 | 0.426 | 0.399 | 0.318 | 0.333 | 0.357 |
| Male group | 0.000 | 0.000 | 0.000 | 0.000 | 0.044 | 0.048 | 0.052 | 0.047 |
| Female group | 0.029 | 0.032 | 0.050 | 0.033 | 0.000 | 0.000 | 0.000 | 0.000 |
| Mixed group | 0.318 | 0.212 | 0.290 | 0.282 | 0.337 | 0.208 | 0.304 | 0.283 |
| Companionship while drinking |  |  |  |  |  |  |  |  |
| With family | 0.292 | 0.164 | 0.182 | 0.235 | 0.252 | 0.124 | 0.161 | 0.188 |
| With friends | 0.150 | 0.122 | 0.237 | 0.157 | 0.159 | 0.127 | 0.215 | 0.158 |
| With partner | 0.531 | 0.471 | 0.484 | 0.505 | 0.527 | 0.406 | 0.462 | 0.470 |
| With other | 0.013 | 0.015 | 0.021 | 0.015 | 0.030 | 0.017 | 0.025 | 0.024 |
| Mood of the occasion |  |  |  |  |  |  |  |  |
| Wind down | 0.372 | 0.458 | 0.385 | 0.400 | 0.311 | 0.386 | 0.319 | 0.340 |
| To have time for themselves | 0.206 | 0.164 | 0.147 | 0.183 | 0.184 | 0.177 | 0.194 | 0.183 |
| To treat/reward themselves | 0.097 | 0.105 | 0.099 | 0.100 | 0.081 | 0.085 | 0.071 | 0.080 |
| To have a break | 0.033 | 0.050 | 0.069 | 0.045 | 0.050 | 0.057 | 0.054 | 0.053 |
| To refresh, recharge or invigorate | 0.062 | 0.066 | 0.053 | 0.062 | 0.112 | 0.114 | 0.146 | 0.119 |
| To bond | 0.066 | 0.042 | 0.084 | 0.062 | 0.071 | 0.041 | 0.088 | 0.063 |
| To let go | 0.028 | 0.031 | 0.044 | 0.032 | 0.039 | 0.051 | 0.047 | 0.045 |
| Have a laugh | 0.052 | 0.051 | 0.099 | 0.060 | 0.058 | 0.050 | 0.100 | 0.063 |
| Other mood | 0.155 | 0.063 | 0.078 | 0.114 | 0.166 | 0.073 | 0.056 | 0.112 |
| Duration of the occasion |  |  |  |  |  |  |  |  |
| Duration (<1 hour) | 0.326 | 0.406 | 0.326 | 0.350 | 0.338 | 0.391 | 0.308 | 0.352 |
| Duration (1-2 hours) | 0.476 | 0.485 | 0.472 | 0.478 | 0.403 | 0.461 | 0.464 | 0.435 |
| Duration (>2 hours) | 0.198 | 0.109 | 0.202 | 0.172 | 0.259 | 0.148 | 0.228 | 0.213 |
| **Table A4 - Discrete variables (continued)** | | | | | | | | |
| **Sample** | **Females** | | | | **Males** | | | |
| **Food choice** | **Meal** | **No food** | **Snack** | **All** | **Meal** | **No food** | **Snack** | **All** |
| Alcohol consumption |  |  |  |  |  |  |  |  |
| 0-2 units | 0.144 | 0.263 | 0.231 | 0.195 | 0.133 | 0.224 | 0.192 | 0.177 |
| 2-3 units | 0.259 | 0.267 | 0.206 | 0.252 | 0.223 | 0.192 | 0.191 | 0.206 |
| 3-5 units | 0.155 | 0.168 | 0.164 | 0.161 | 0.129 | 0.155 | 0.134 | 0.139 |
| 5-12 units | 0.337 | 0.239 | 0.278 | 0.298 | 0.327 | 0.312 | 0.314 | 0.319 |
| 12-20 units | 0.068 | 0.041 | 0.078 | 0.062 | 0.108 | 0.078 | 0.106 | 0.097 |
| > 20 units | 0.037 | 0.021 | 0.043 | 0.034 | 0.081 | 0.038 | 0.063 | 0.062 |
| Main drinking beverage |  |  |  |  |  |  |  |  |
| RTD | 0.014 | 0.018 | 0.023 | 0.017 | 0.008 | 0.008 | 0.008 | 0.008 |
| Spirits | 0.155 | 0.297 | 0.260 | 0.215 | 0.143 | 0.265 | 0.240 | 0.206 |
| Cider | 0.079 | 0.085 | 0.108 | 0.086 | 0.109 | 0.123 | 0.146 | 0.121 |
| Wine | 0.654 | 0.442 | 0.463 | 0.557 | 0.396 | 0.145 | 0.165 | 0.260 |
| Beer | 0.098 | 0.157 | 0.146 | 0.124 | 0.344 | 0.459 | 0.441 | 0.404 |
| Start time of the drinking occasion |  |  |  |  |  |  |  |  |
| Morning | 0.005 | 0.013 | 0.014 | 0.009 | 0.013 | 0.033 | 0.024 | 0.022 |
| Lunch | 0.059 | 0.020 | 0.050 | 0.046 | 0.077 | 0.045 | 0.075 | 0.064 |
| Afternoon | 0.161 | 0.129 | 0.120 | 0.144 | 0.196 | 0.127 | 0.142 | 0.161 |
| Evening | 0.761 | 0.714 | 0.732 | 0.742 | 0.687 | 0.629 | 0.645 | 0.658 |
| Night time | 0.015 | 0.124 | 0.084 | 0.059 | 0.027 | 0.166 | 0.115 | 0.095 |
| Day of the week |  |  |  |  |  |  |  |  |
| Weekdays | 0.375 | 0.409 | 0.359 | 0.382 | 0.402 | 0.440 | 0.411 | 0.418 |
| Weekend | 0.421 | 0.441 | 0.488 | 0.439 | 0.401 | 0.415 | 0.421 | 0.410 |
| Sunday | 0.204 | 0.150 | 0.153 | 0.179 | 0.197 | 0.145 | 0.168 | 0.172 |
| Type of the occasion (off-trade) |  |  |  |  |  |  |  |  |
| Socialize | 0.162 | 0.107 | 0.210 | 0.154 | 0.138 | 0.097 | 0.188 | 0.132 |
| Regular drink | 0.335 | 0.274 | 0.253 | 0.302 | 0.376 | 0.288 | 0.302 | 0.330 |
| Night cap | 0.038 | 0.145 | 0.111 | 0.082 | 0.039 | 0.130 | 0.092 | 0.082 |
| Quiet drink | 0.353 | 0.388 | 0.327 | 0.359 | 0.301 | 0.377 | 0.343 | 0.337 |
| Other type | 0.113 | 0.070 | 0.082 | 0.095 | 0.145 | 0.095 | 0.085 | 0.115 |
| Off-trade location |  |  |  |  |  |  |  |  |
| Own home | 0.849 | 0.874 | 0.801 | 0.848 | 0.849 | 0.886 | 0.843 | 0.861 |
| Someone else’s home | 0.141 | 0.100 | 0.181 | 0.136 | 0.126 | 0.086 | 0.132 | 0.112 |
| Other off-trade location | 0.037 | 0.037 | 0.049 | 0.040 | 0.050 | 0.038 | 0.048 | 0.045 |
| Activities while drinking (off-trade only) | | | | |  |  |  |  |
| Watching TV | 0.521 | 0.606 | 0.605 | 0.561 | 0.526 | 0.579 | 0.576 | 0.555 |
| Internet-related activity | 0.219 | 0.194 | 0.260 | 0.219 | 0.247 | 0.236 | 0.306 | 0.254 |
| Games and leisure | 0.198 | 0.139 | 0.212 | 0.183 | 0.225 | 0.154 | 0.272 | 0.208 |
| Chores | 0.270 | 0.073 | 0.086 | 0.179 | 0.240 | 0.045 | 0.074 | 0.137 |
| Get ready | 0.009 | 0.012 | 0.018 | 0.011 | 0.014 | 0.013 | 0.016 | 0.014 |
| N | 7,570 | 4,406 | 2,708 | 14,684 | 9,269 | 7,934 | 4,071 | 21,274 |

**Table A4 - Summary statistics by food choice (meal consumed, snack only, no food) and gender. On-trade occasions. Continuous variables**

| **Sample** | **Females** | | | | | **Males** | | | | |
| --- | --- | --- | --- | --- | --- | --- | --- | --- | --- | --- |
|  | **N** | **Mean** | **SD** | **Min** | **Max** | **N** | **Mean** | **SD** | **Min** | **Max** |
| **Food choice: meal** |  |  |  |  |  |  |  |  |  |  |
| age | 1,934 | 46.38 | 17.32 | 18 | 80 | 4,464 | 47.69 | 16.79 | 18 | 82 |
| units | 1,934 | 5.82 | 6.45 | 0.16 | 40 | 4,464 | 8.69 | 7.34 | 0.28 | 40 |
| **Food choice: no food** |  |  |  |  |  |  |  |  |  |  |
| age | 1,987 | 43.38 | 17.64 | 18 | 87 | 649 | 42.64 | 16.66 | 18 | 85 |
| units | 1,987 | 6.33 | 6.30 | 0.16 | 40 | 649 | 9.26 | 7.98 | 0.92 | 40 |
| **Food choice: snack** |  |  |  |  |  |  |  |  |  |  |
| age | 486 | 44.64 | 17.42 | 18 | 78 | 2,167 | 46.95 | 17.72 | 18 | 90 |
| units | 486 | 6.34 | 7.20 | 0.16 | 40 | 2,167 | 7.58 | 7.85 | 0.03 | 40 |
| **Food choice: all** |  |  |  |  |  |  |  |  |  |  |
| age | 4,407 | 44.91 | 17.52 | 18 | 87 | 21,274 | 49.59 | 16.51 | 18 | 90 |
| units | 4,407 | 6.09 | 6.48 | 0.16 | 40 | 21,274 | 7.11 | 7.11 | 0.13 | 40 |

| **Discrete variables** |  | | | |  | | | | |
| --- | --- | --- | --- | --- | --- | --- | --- | --- | --- |
| **Sample** | **Females** | | | | | **Males** | | | |
| **Food choice** | **Meal** | **No food** | **Snack** | **All** | | **Meal** | **No food** | **Snack** | **All** |
| Individual characteristics |  |  |  |  | |  |  |  |  |
| Social Class AB | 0.275 | 0.219 | 0.256 | 0.249 | | 0.361 | 0.270 | 0.298 | 0.299 |
| Social Class C1 | 0.299 | 0.365 | 0.276 | 0.325 | | 0.287 | 0.313 | 0.337 | 0.308 |
| Social Class C2 | 0.248 | 0.222 | 0.247 | 0.237 | | 0.211 | 0.217 | 0.215 | 0.215 |
| Social Class DE | 0.178 | 0.194 | 0.221 | 0.189 | | 0.140 | 0.200 | 0.150 | 0.178 |
| Household Income (< 20k) | 0.240 | 0.245 | 0.305 | 0.249 | | 0.185 | 0.269 | 0.198 | 0.238 |
| Household Income (20k - 35k) | 0.292 | 0.283 | 0.280 | 0.287 | | 0.287 | 0.300 | 0.283 | 0.294 |
| Household Income (35k - 55k) | 0.246 | 0.215 | 0.184 | 0.226 | | 0.276 | 0.217 | 0.279 | 0.240 |
| Household Income (> 55k) | 0.101 | 0.141 | 0.110 | 0.119 | | 0.171 | 0.144 | 0.159 | 0.153 |
| Full-time worker | 0.359 | 0.449 | 0.414 | 0.404 | | 0.546 | 0.556 | 0.628 | 0.559 |
| Other employment status | 0.641 | 0.551 | 0.586 | 0.596 | | 0.454 | 0.444 | 0.372 | 0.441 |
| Married | 0.379 | 0.430 | 0.428 | 0.406 | | 0.345 | 0.497 | 0.474 | 0.450 |
| Children in the household binary | 0.250 | 0.225 | 0.246 | 0.239 | | 0.236 | 0.176 | 0.219 | 0.197 |
| Gender composition of the drinking occasion | | | | | |  |  |  |  |
| Male alone | 0.000 | 0.000 | 0.000 | 0.000 | | 0.047 | 0.156 | 0.094 | 0.119 |
| Female alone | 0.009 | 0.042 | 0.023 | 0.025 | | 0.000 | 0.000 | 0.000 | 0.000 |
| Male pair | 0.000 | 0.000 | 0.000 | 0.000 | | 0.085 | 0.129 | 0.136 | 0.117 |
| Female pair | 0.118 | 0.082 | 0.080 | 0.098 | | 0.000 | 0.000 | 0.000 | 0.000 |
| Mixed pair | 0.300 | 0.254 | 0.255 | 0.276 | | 0.296 | 0.097 | 0.118 | 0.157 |
| Male group | 0.000 | 0.000 | 0.000 | 0.000 | | 0.131 | 0.303 | 0.237 | 0.246 |
| Female group | 0.115 | 0.108 | 0.151 | 0.116 | | 0.000 | 0.000 | 0.000 | 0.000 |
| Mixed group | 0.495 | 0.553 | 0.539 | 0.524 | | 0.492 | 0.360 | 0.488 | 0.410 |
| Companionship while drinking |  |  |  |  | |  |  |  |  |
| With family | 0.346 | 0.138 | 0.223 | 0.244 | | 0.282 | 0.096 | 0.203 | 0.160 |
| With friends | 0.378 | 0.534 | 0.507 | 0.459 | | 0.396 | 0.564 | 0.586 | 0.517 |
| With partner | 0.419 | 0.396 | 0.382 | 0.405 | | 0.429 | 0.170 | 0.210 | 0.250 |
| With other | 0.080 | 0.097 | 0.118 | 0.091 | | 0.101 | 0.115 | 0.115 | 0.111 |
| Mood of the occasion |  |  |  |  | |  |  |  |  |
| Wind down | 0.104 | 0.153 | 0.139 | 0.129 | | 0.100 | 0.151 | 0.188 | 0.139 |
| To have time for themselves | 0.300 | 0.153 | 0.192 | 0.226 | | 0.253 | 0.133 | 0.134 | 0.169 |
| To treat/reward themselves | 0.078 | 0.048 | 0.041 | 0.061 | | 0.068 | 0.047 | 0.036 | 0.052 |
| To have a break | 0.085 | 0.085 | 0.104 | 0.087 | | 0.088 | 0.105 | 0.079 | 0.098 |
| To refresh, recharge or invigorate | 0.045 | 0.096 | 0.076 | 0.070 | | 0.091 | 0.105 | 0.096 | 0.100 |
| To bond | 0.211 | 0.204 | 0.181 | 0.205 | | 0.208 | 0.222 | 0.279 | 0.223 |
| To let go | 0.018 | 0.037 | 0.042 | 0.029 | | 0.029 | 0.047 | 0.050 | 0.042 |
| Have a laugh | 0.100 | 0.179 | 0.190 | 0.143 | | 0.109 | 0.177 | 0.164 | 0.156 |
| Other mood | 0.126 | 0.104 | 0.100 | 0.114 | | 0.125 | 0.082 | 0.067 | 0.093 |
| Duration of the occasion |  |  |  |  | |  |  |  |  |
| Duration (<1 hour) | 0.065 | 0.175 | 0.118 | 0.118 | | 0.106 | 0.183 | 0.109 | 0.154 |
| Duration (1-2 hours) | 0.666 | 0.528 | 0.516 | 0.591 | | 0.615 | 0.508 | 0.547 | 0.543 |
| Duration (>2 hours) | 0.269 | 0.298 | 0.366 | 0.292 | | 0.279 | 0.309 | 0.343 | 0.303 |
| **Table A4 - Discrete variables (continued)** | | | | | | | | | |
| **Sample** | **Females** | | | | | **Males** | | | |
| **Food choice** | **Meal** | **No food** | **Snack** | **All** | | **Meal** | **No food** | **Snack** | **All** |
| Alcohol consumption |  |  |  |  | |  |  |  |  |
| 0-2 units | 0.144 | 0.148 | 0.158 | 0.147 | | 0.137 | 0.066 | 0.064 | 0.087 |
| 2-3 units | 0.367 | 0.272 | 0.334 | 0.322 | | 0.275 | 0.185 | 0.170 | 0.210 |
| 3-5 units | 0.145 | 0.150 | 0.154 | 0.148 | | 0.127 | 0.145 | 0.152 | 0.140 |
| 5-12 units | 0.240 | 0.309 | 0.215 | 0.267 | | 0.271 | 0.386 | 0.353 | 0.350 |
| 12-20 units | 0.060 | 0.077 | 0.073 | 0.069 | | 0.112 | 0.140 | 0.175 | 0.135 |
| > 20 units | 0.044 | 0.044 | 0.065 | 0.046 | | 0.077 | 0.078 | 0.086 | 0.078 |
| Main drinking beverage |  |  |  |  | |  |  |  |  |
| RTD | 0.026 | 0.034 | 0.040 | 0.031 | | 0.013 | 0.018 | 0.021 | 0.017 |
| Spirits | 0.170 | 0.293 | 0.228 | 0.229 | | 0.084 | 0.104 | 0.118 | 0.100 |
| Cider | 0.135 | 0.152 | 0.150 | 0.144 | | 0.119 | 0.105 | 0.131 | 0.111 |
| Wine | 0.465 | 0.228 | 0.262 | 0.341 | | 0.153 | 0.036 | 0.053 | 0.072 |
| Beer | 0.205 | 0.292 | 0.320 | 0.255 | | 0.631 | 0.737 | 0.677 | 0.700 |
| Start time of the drinking occasion |  |  |  |  | |  |  |  |  |
| Morning | 0.033 | 0.030 | 0.035 | 0.032 | | 0.042 | 0.047 | 0.051 | 0.046 |
| Lunch | 0.266 | 0.102 | 0.148 | 0.183 | | 0.236 | 0.135 | 0.125 | 0.164 |
| Afternoon | 0.241 | 0.221 | 0.233 | 0.232 | | 0.227 | 0.254 | 0.222 | 0.243 |
| Evening | 0.449 | 0.553 | 0.560 | 0.505 | | 0.474 | 0.471 | 0.549 | 0.479 |
| Night time | 0.011 | 0.095 | 0.023 | 0.048 | | 0.021 | 0.093 | 0.054 | 0.068 |
| Day of the week |  |  |  |  | |  |  |  |  |
| Weekdays | 0.428 | 0.356 | 0.394 | 0.394 | | 0.425 | 0.412 | 0.372 | 0.412 |
| Weekend | 0.400 | 0.503 | 0.442 | 0.449 | | 0.406 | 0.438 | 0.455 | 0.430 |
| Sunday | 0.172 | 0.141 | 0.164 | 0.158 | | 0.170 | 0.150 | 0.174 | 0.158 |
| On-trade venue |  |  |  |  | |  |  |  |  |
| Club | 0.021 | 0.094 | 0.053 | 0.056 | | 0.030 | 0.058 | 0.068 | 0.050 |
| Modern bar | 0.104 | 0.189 | 0.170 | 0.148 | | 0.135 | 0.168 | 0.181 | 0.160 |
| Traditional pub | 0.158 | 0.377 | 0.382 | 0.275 | | 0.203 | 0.458 | 0.393 | 0.377 |
| Family pub | 0.111 | 0.081 | 0.092 | 0.096 | | 0.099 | 0.072 | 0.094 | 0.082 |
| Food pub | 0.342 | 0.172 | 0.172 | 0.251 | | 0.321 | 0.159 | 0.165 | 0.207 |
| Student pub | 0.008 | 0.014 | 0.008 | 0.011 | | 0.015 | 0.012 | 0.018 | 0.014 |
| Social club | 0.012 | 0.054 | 0.038 | 0.033 | | 0.018 | 0.097 | 0.054 | 0.070 |
| Restaurant | 0.299 | 0.028 | 0.068 | 0.158 | | 0.276 | 0.024 | 0.065 | 0.102 |
| Other on-trade venue | 0.146 | 0.127 | 0.176 | 0.141 | | 0.111 | 0.105 | 0.140 | 0.110 |
| Activities while drinking (on-trade only) | | | | | |  |  |  |  |
| Watching TV | 0.051 | 0.064 | 0.084 | 0.060 | | 0.096 | 0.162 | 0.214 | 0.147 |
| Game quiz | 0.024 | 0.044 | 0.061 | 0.036 | | 0.055 | 0.080 | 0.116 | 0.076 |
| Pub quiz | 0.015 | 0.049 | 0.033 | 0.032 | | 0.018 | 0.030 | 0.038 | 0.027 |
| Active game | 0.027 | 0.070 | 0.085 | 0.052 | | 0.059 | 0.089 | 0.141 | 0.085 |
| Music game | 0.016 | 0.043 | 0.052 | 0.031 | | 0.025 | 0.046 | 0.054 | 0.040 |
| Live music | 0.080 | 0.176 | 0.234 | 0.138 | | 0.102 | 0.132 | 0.173 | 0.127 |
| Drink outside | 0.098 | 0.158 | 0.172 | 0.132 | | 0.086 | 0.107 | 0.155 | 0.105 |
| Other on-trade activity | 0.080 | 0.111 | 0.108 | 0.096 | | 0.098 | 0.093 | 0.113 | 0.096 |
| N | 1,934 | 1,987 | 486 | 4,407 | | 2,167 | 4,464 | 649 | 7,280 |

1. The definition of drinking occasion used in the present analysis is based on time interval between subsequent drinks. If the respondent reports the time of a drink to start more than two hours after the reported end of a previous drink, these are considered two different drinking occasions. If the gap between drinks is smaller than two hours, they belong to the same drinking occasion. [↑](#endnote-ref-1)
2. We compute sampling weights to adjust for potential sample bias. In particular, we used the method of raking to adjust for differences between the observed sample characteristics (age, gender, social grade, and region) and the UK Census population. [↑](#endnote-ref-2)
3. As robustness check, we test for statistically significant differences across samples. In this case, testing for statistical significant difference is quite challenging due to the multilevel nature of the data (i.e. occasion-level data nested within individuals). To tackle this problem, we estimate a multinomial logit model where the dependent variable is represented by the type of food consumed (meal, no food, or snacks), and the set of explanatory variables are the occasion and individual characteristics described above. In addition, we add an individual random effect to account for the different levels of information. [↑](#endnote-ref-3)
